# 4-[(2′-Cyano­biphen­yl-4-yl)meth­yl]morpholin-4-ium tetra­fluoridoborate

**DOI:** 10.1107/S160053681101186X

**Published:** 2011-04-07

**Authors:** Xiu-juan Li, Xian-gang Huang, Kang-jun Li

**Affiliations:** a College of Food Engineering, RiZhao Polytechnic, RiZhao 276826, People’s Republic of China

## Abstract

In the crystal structure of the title compound, C_18_H_19_N_2_O^+^·BF_4_^−^, bifurcated N—H⋯(F,F) hydrogen bonds link the protonated 4′-morpholine­methyl­biphenyl-2-carbonitrile cations and slightly distorted tetra­fluoro­borate anions. π–π inter­actions [centroid–centroid distance = 3.805 (3) Å] help to consolidate the packing. The dihedral angle between the benzene rings in the cation is 57.24 (11)°.

## Related literature

For a related structure, see: SiMa (2010 ▶).
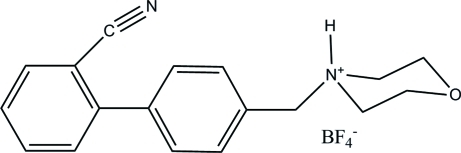


## Experimental

### 

#### Crystal data

C_18_H_19_N_2_O^+^·BF_4_^−^
*M* *_r_* = 366.16
Triclinic, 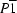

*a* = 9.059 (6) Å
*b* = 9.859 (8) Å
*c* = 10.597 (8) Å
α = 76.324 (14)°
β = 83.71 (2)°
γ = 86.50 (3)°
*V* = 913.5 (12) Å^3^
*Z* = 2
Mo *K*α radiation
μ = 0.11 mm^−1^
*T* = 298 K
0.20 × 0.20 × 0.20 mm

#### Data collection

Rigaku SCXmini diffractometer
Absorption correction: multi-scan (*CrystalClear*; Rigaku, 2005 ▶) *T*_min_ = 0.978, *T*_max_ = 0.978
9817 measured reflections
4098 independent reflections
3039 reflections with *I* > 2σ(*I*)
*R*_int_ = 0.028

#### Refinement

*R*[*F* ^2^ > 2σ(*F* ^2^)] = 0.067
*wR*(*F* ^2^) = 0.202
*S* = 1.06
4098 reflections
239 parameters
1 restraint
H atoms treated by a mixture of independent and constrained refinement
Δρ_max_ = 0.49 e Å^−3^
Δρ_min_ = −0.30 e Å^−3^

### 

Data collection: *CrystalClear* (Rigaku, 2005 ▶); cell refinement: *CrystalClear*; data reduction: *CrystalClear*; program(s) used to solve structure: *SHELXS97* (Sheldrick, 2008 ▶); program(s) used to refine structure: *SHELXL97* (Sheldrick, 2008 ▶); molecular graphics: *SHELXTL* (Sheldrick, 2008 ▶); software used to prepare material for publication: *SHELXTL*.

## Supplementary Material

Crystal structure: contains datablocks I, global. DOI: 10.1107/S160053681101186X/jh2276sup1.cif

Structure factors: contains datablocks I. DOI: 10.1107/S160053681101186X/jh2276Isup2.hkl

Additional supplementary materials:  crystallographic information; 3D view; checkCIF report

## Figures and Tables

**Table 1:** Hydrogen-bond geometry (Å, °)

*D*—H⋯*A*	*D*—H	H⋯*A*	*D*⋯*A*	*D*—H⋯*A*
N2—H2⋯F1	0.90 (2)	2.14 (2)	2.902 (3)	141 (2)
N2—H2⋯F3	0.90 (2)	2.35 (2)	3.219 (3)	161 (2)

**Fig. 1.**
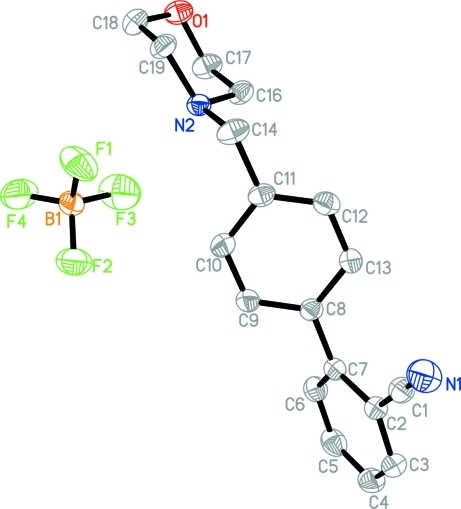
The molecular structure of the title compound, with the atomic numbering scheme. Displacement ellipsoids are drawn at the 30% probability level.

**Fig. 2.**
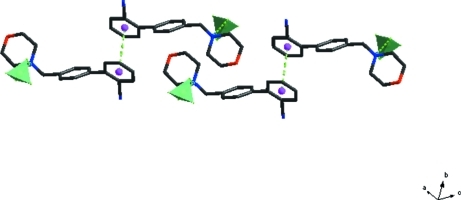
A view of the packing of the title compound. Dashed lines indicate hydrogen bonds and π-π interactions.

## supplementary crystallographic information

### Comment

The crystal structure of 4'-morpholinemethylbiphenyl-2-carbonitrile with nitrate is known (SiMa, 2010).

The asymmetric unit of the title compound is built up of one 4'-morpholinemethylbiphenyl-2-carbonitrile cation with the dihedral angle of 57.24 (11)° between two benzene rings and one tetrafluoroborate anion (Fig 1). The intermolecular N—H···F hydrogen bonds link the cations and anions to chains (Table 1). The π–π stacking interactions of adjacent cyanobenzene rings with a centroid–centroid distance of 3.805 (3)Å stabilize the crystal structure (Fig 2).

### Experimental

Tetrafluoroboric acid(10 mmol) was added dropwise under stirring to a solution of 4'-morpholinemethylbiphenyl-2-carbonitrile (10 mmol) ethanol solution. Water was added until all suspended substrates disappeared. Colorless single crystals of the title compound suitable for X-ray analysis were obtained by slow evaporation from the solution at room temperature after 5 d, giving a yield of *ca* 78%.

### Refinement

Positional parameters of all the H atoms for C atoms were calculated geometrically and were allowed to ride on the C atoms to which they are bonded, with *U*_iso_(H) = 1.2Ueq(C). The H atoms bonded to N atoms were found in a difference Fourier map and refined with restraints for N—H distances of 0.87 (2).

### Crystal data

**Table:**

C_18_H_19_N_2_O^+^·BF_4_^−^	*Z* = 2
*M**_r_* = 366.16	*F*(000) = 380
Triclinic, *P*1	*D*_x_ = 1.331 Mg m^−^^3^
Hall symbol: -P 1	Mo *K*α radiation, λ = 0.71073 Å
*a* = 9.059 (6) Å	Cell parameters from 2199 reflections
*b* = 9.859 (8) Å	θ = 2.6–27.4°
*c* = 10.597 (8) Å	µ = 0.11 mm^−^^1^
α = 76.324 (14)°	*T* = 298 K
β = 83.71 (2)°	Prism, colourless
γ = 86.50 (3)°	0.20 × 0.20 × 0.20 mm
*V* = 913.5 (12) Å^3^	

### Data collection

**Table:**

Rigaku SCXmini diffractometer	4098 independent reflections
Radiation source: fine-focus sealed tube	3039 reflections with *I* > 2σ(*I*)
graphite	*R*_int_ = 0.028
Detector resolution: 13.6612 pixels mm^-1^	θ_max_ = 27.4°, θ_min_ = 2.6°
ω scans	*h* = −11→11
Absorption correction: multi-scan (*CrystalClear*; Rigaku, 2005)	*k* = −12→12
*T*_min_ = 0.978, *T*_max_ = 0.978	*l* = −13→13
9817 measured reflections	

### Refinement

**Table:**

Refinement on *F*^2^	Primary atom site location: structure-invariant direct methods
Least-squares matrix: full	Secondary atom site location: difference Fourier map
*R*[*F*^2^ > 2σ(*F*^2^)] = 0.067	Hydrogen site location: inferred from neighbouring sites
*wR*(*F*^2^) = 0.202	H atoms treated by a mixture of independent and constrained refinement
*S* = 1.06	*w* = 1/[σ^2^(*F*_o_^2^) + (0.1049*P*)^2^ + 0.2107*P*] where *P* = (*F*_o_^2^ + 2*F*_c_^2^)/3
4098 reflections	(Δ/σ)_max_ < 0.001
239 parameters	Δρ_max_ = 0.49 e Å^−^^3^
1 restraint	Δρ_min_ = −0.30 e Å^−^^3^

### Special details

**Table:**

Geometry. All e.s.d.'s (except the e.s.d. in the dihedral angle between two l.s. planes) are estimated using the full covariance matrix. The cell e.s.d.'s are taken into account individually in the estimation of e.s.d.'s in distances, angles and torsion angles; correlations between e.s.d.'s in cell parameters are only used when they are defined by crystal symmetry. An approximate (isotropic) treatment of cell e.s.d.'s is used for estimating e.s.d.'s involving l.s. planes.
Refinement. Refinement of *F*^2^ against ALL reflections. The weighted *R*-factor *wR* and goodness of fit *S* are based on *F*^2^, conventional *R*-factors *R* are based on *F*, with *F* set to zero for negative *F*^2^. The threshold expression of *F*^2^ > σ(*F*^2^) is used only for calculating *R*-factors(gt) *etc*. and is not relevant to the choice of reflections for refinement. *R*-factors based on *F*^2^ are statistically about twice as large as those based on *F*, and *R*- factors based on ALL data will be even larger.

### Fractional atomic coordinates and isotropic or equivalent isotropic displacement parameters (Å^2^)

**Table:**

	*x*	*y*	*z*	*U*_iso_*/*U*_eq_	
C1	0.9918 (2)	0.3886 (3)	0.3969 (2)	0.0559 (5)	
C2	1.0478 (2)	0.2599 (2)	0.47565 (19)	0.0472 (5)	
C3	1.1901 (2)	0.2084 (3)	0.4395 (2)	0.0613 (6)	
H3A	1.2463	0.2570	0.3658	0.074*	
C4	1.2460 (3)	0.0863 (3)	0.5129 (3)	0.0662 (7)	
H4A	1.3404	0.0522	0.4890	0.079*	
C5	1.1631 (3)	0.0144 (3)	0.6214 (2)	0.0644 (6)	
H5A	1.2009	−0.0692	0.6700	0.077*	
C6	1.0234 (2)	0.0653 (2)	0.6593 (2)	0.0533 (5)	
H6A	0.9695	0.0162	0.7341	0.064*	
C7	0.9623 (2)	0.1888 (2)	0.58744 (18)	0.0436 (4)	
C8	0.8154 (2)	0.24449 (19)	0.63377 (18)	0.0424 (4)	
C9	0.7937 (2)	0.2673 (2)	0.75930 (19)	0.0521 (5)	
H9A	0.8707	0.2464	0.8128	0.062*	
C10	0.6601 (2)	0.3203 (3)	0.8053 (2)	0.0564 (5)	
H10A	0.6487	0.3376	0.8885	0.068*	
C11	0.5416 (2)	0.3483 (2)	0.7281 (2)	0.0475 (5)	
C12	0.5617 (2)	0.3237 (2)	0.6039 (2)	0.0513 (5)	
H12A	0.4831	0.3409	0.5519	0.062*	
C13	0.6973 (2)	0.2736 (2)	0.55624 (19)	0.0480 (5)	
H13A	0.7098	0.2594	0.4720	0.058*	
C14	0.3952 (2)	0.4050 (2)	0.7809 (2)	0.0570 (6)	
H14A	0.4119	0.4897	0.8081	0.068*	
H14B	0.3293	0.4291	0.7120	0.068*	
C16	0.2802 (2)	0.1734 (2)	0.8569 (2)	0.0523 (5)	
H16A	0.3689	0.1267	0.8251	0.063*	
H16B	0.2158	0.1995	0.7870	0.063*	
C17	0.2012 (3)	0.0752 (2)	0.9722 (3)	0.0633 (6)	
H17A	0.1733	−0.0066	0.9457	0.076*	
H17B	0.2678	0.0446	1.0400	0.076*	
C18	0.1109 (3)	0.2580 (3)	1.0663 (2)	0.0645 (6)	
H18A	0.1768	0.2267	1.1345	0.077*	
H18B	0.0222	0.3002	1.1034	0.077*	
C19	0.1868 (2)	0.3662 (2)	0.9572 (2)	0.0550 (5)	
H19A	0.1184	0.4039	0.8922	0.066*	
H19B	0.2162	0.4423	0.9914	0.066*	
N1	0.9501 (3)	0.4903 (3)	0.3325 (2)	0.0805 (7)	
N2	0.32178 (17)	0.30144 (17)	0.89484 (16)	0.0432 (4)	
H2	0.384 (2)	0.273 (3)	0.958 (2)	0.064 (7)*	
O1	0.07139 (17)	0.14212 (18)	1.02265 (16)	0.0638 (4)	
B1	0.5558 (3)	0.2295 (3)	1.1710 (3)	0.0583 (6)	
F1	0.4631 (2)	0.33813 (19)	1.1168 (2)	0.1044 (7)	
F2	0.70030 (18)	0.2643 (2)	1.13544 (18)	0.0962 (6)	
F3	0.5250 (3)	0.1264 (2)	1.1129 (3)	0.1298 (9)	
F4	0.5317 (2)	0.2022 (4)	1.29990 (17)	0.1466 (12)	

### Atomic displacement parameters (Å^2^)

**Table:**

	*U*^11^	*U*^22^	*U*^33^	*U*^12^	*U*^13^	*U*^23^
C1	0.0508 (12)	0.0678 (14)	0.0446 (11)	−0.0121 (10)	−0.0021 (9)	−0.0030 (10)
C2	0.0432 (10)	0.0557 (11)	0.0435 (10)	−0.0053 (8)	−0.0042 (8)	−0.0122 (8)
C3	0.0464 (12)	0.0853 (16)	0.0557 (12)	−0.0068 (11)	0.0029 (9)	−0.0257 (12)
C4	0.0478 (12)	0.0833 (17)	0.0756 (16)	0.0131 (11)	−0.0107 (11)	−0.0360 (14)
C5	0.0664 (14)	0.0620 (13)	0.0698 (15)	0.0143 (11)	−0.0248 (12)	−0.0215 (12)
C6	0.0572 (12)	0.0519 (11)	0.0503 (11)	0.0005 (9)	−0.0105 (9)	−0.0090 (9)
C7	0.0411 (10)	0.0486 (10)	0.0421 (10)	−0.0043 (8)	−0.0064 (7)	−0.0107 (8)
C8	0.0398 (9)	0.0442 (10)	0.0409 (9)	−0.0062 (7)	−0.0018 (7)	−0.0049 (8)
C9	0.0424 (10)	0.0708 (13)	0.0423 (10)	−0.0109 (9)	−0.0033 (8)	−0.0099 (9)
C10	0.0463 (11)	0.0756 (15)	0.0490 (11)	−0.0132 (10)	0.0053 (8)	−0.0196 (10)
C11	0.0411 (10)	0.0427 (10)	0.0556 (11)	−0.0074 (8)	0.0039 (8)	−0.0077 (8)
C12	0.0441 (10)	0.0528 (11)	0.0541 (11)	−0.0020 (8)	−0.0094 (8)	−0.0047 (9)
C13	0.0476 (11)	0.0542 (11)	0.0423 (10)	−0.0019 (8)	−0.0062 (8)	−0.0106 (8)
C14	0.0495 (11)	0.0417 (10)	0.0723 (14)	−0.0059 (8)	0.0111 (10)	−0.0054 (10)
C16	0.0475 (11)	0.0501 (11)	0.0614 (12)	−0.0106 (8)	0.0070 (9)	−0.0208 (10)
C17	0.0554 (13)	0.0523 (12)	0.0777 (15)	−0.0121 (10)	0.0077 (11)	−0.0101 (11)
C18	0.0563 (13)	0.0836 (16)	0.0521 (12)	−0.0033 (11)	0.0075 (10)	−0.0183 (12)
C19	0.0485 (11)	0.0586 (12)	0.0594 (12)	0.0031 (9)	0.0049 (9)	−0.0224 (10)
N1	0.0768 (15)	0.0817 (15)	0.0685 (13)	−0.0147 (12)	−0.0147 (11)	0.0177 (12)
N2	0.0370 (8)	0.0448 (8)	0.0486 (9)	−0.0033 (6)	−0.0026 (7)	−0.0125 (7)
O1	0.0457 (8)	0.0748 (11)	0.0671 (10)	−0.0151 (7)	0.0091 (7)	−0.0123 (8)
B1	0.0525 (14)	0.0645 (15)	0.0594 (15)	0.0077 (11)	−0.0145 (11)	−0.0158 (12)
F1	0.1023 (14)	0.0844 (12)	0.1375 (16)	0.0327 (10)	−0.0665 (12)	−0.0316 (11)
F2	0.0606 (10)	0.1263 (15)	0.0970 (12)	−0.0091 (9)	−0.0087 (8)	−0.0145 (11)
F3	0.1141 (16)	0.0880 (13)	0.211 (3)	−0.0028 (11)	−0.0255 (16)	−0.0766 (16)
F4	0.0801 (13)	0.281 (3)	0.0580 (10)	0.0229 (16)	−0.0057 (8)	−0.0060 (14)

### Geometric parameters (Å, °)

**Table:**

C1—N1	1.140 (3)	C13—H13A	0.9300
C1—C2	1.441 (3)	C14—N2	1.506 (3)
C2—C7	1.403 (3)	C14—H14A	0.9700
C2—C3	1.403 (3)	C14—H14B	0.9700
C3—C4	1.372 (4)	C16—N2	1.493 (3)
C3—H3A	0.9300	C16—C17	1.509 (3)
C4—C5	1.371 (4)	C16—H16A	0.9700
C4—H4A	0.9300	C16—H16B	0.9700
C5—C6	1.386 (3)	C17—O1	1.428 (3)
C5—H5A	0.9300	C17—H17A	0.9700
C6—C7	1.393 (3)	C17—H17B	0.9700
C6—H6A	0.9300	C18—O1	1.408 (3)
C7—C8	1.486 (3)	C18—C19	1.510 (3)
C8—C9	1.392 (3)	C18—H18A	0.9700
C8—C13	1.395 (3)	C18—H18B	0.9700
C9—C10	1.374 (3)	C19—N2	1.505 (3)
C9—H9A	0.9300	C19—H19A	0.9700
C10—C11	1.396 (3)	C19—H19B	0.9700
C10—H10A	0.9300	N2—H2	0.904 (16)
C11—C12	1.386 (3)	B1—F4	1.324 (3)
C11—C14	1.509 (3)	B1—F3	1.363 (3)
C12—C13	1.384 (3)	B1—F2	1.363 (3)
C12—H12A	0.9300	B1—F1	1.374 (3)
			
N1—C1—C2	178.5 (3)	N2—C14—H14B	109.2
C7—C2—C3	120.7 (2)	C11—C14—H14B	109.2
C7—C2—C1	120.47 (18)	H14A—C14—H14B	107.9
C3—C2—C1	118.78 (19)	N2—C16—C17	110.42 (18)
C4—C3—C2	120.0 (2)	N2—C16—H16A	109.6
C4—C3—H3A	120.0	C17—C16—H16A	109.6
C2—C3—H3A	120.0	N2—C16—H16B	109.6
C5—C4—C3	120.1 (2)	C17—C16—H16B	109.6
C5—C4—H4A	120.0	H16A—C16—H16B	108.1
C3—C4—H4A	120.0	O1—C17—C16	110.81 (19)
C4—C5—C6	120.5 (2)	O1—C17—H17A	109.5
C4—C5—H5A	119.8	C16—C17—H17A	109.5
C6—C5—H5A	119.8	O1—C17—H17B	109.5
C5—C6—C7	121.3 (2)	C16—C17—H17B	109.5
C5—C6—H6A	119.4	H17A—C17—H17B	108.1
C7—C6—H6A	119.4	O1—C18—C19	111.95 (18)
C6—C7—C2	117.45 (19)	O1—C18—H18A	109.2
C6—C7—C8	120.07 (17)	C19—C18—H18A	109.2
C2—C7—C8	122.40 (18)	O1—C18—H18B	109.2
C9—C8—C13	118.51 (18)	C19—C18—H18B	109.2
C9—C8—C7	119.17 (17)	H18A—C18—H18B	107.9
C13—C8—C7	122.31 (18)	N2—C19—C18	110.08 (18)
C10—C9—C8	121.01 (19)	N2—C19—H19A	109.6
C10—C9—H9A	119.5	C18—C19—H19A	109.6
C8—C9—H9A	119.5	N2—C19—H19B	109.6
C9—C10—C11	120.4 (2)	C18—C19—H19B	109.6
C9—C10—H10A	119.8	H19A—C19—H19B	108.2
C11—C10—H10A	119.8	C16—N2—C19	109.84 (16)
C12—C11—C10	118.88 (19)	C16—N2—C14	112.09 (17)
C12—C11—C14	121.43 (19)	C19—N2—C14	111.26 (16)
C10—C11—C14	119.7 (2)	C16—N2—H2	107.1 (16)
C13—C12—C11	120.69 (19)	C19—N2—H2	105.8 (16)
C13—C12—H12A	119.7	C14—N2—H2	110.5 (16)
C11—C12—H12A	119.7	C18—O1—C17	110.10 (17)
C12—C13—C8	120.45 (19)	F4—B1—F3	116.5 (3)
C12—C13—H13A	119.8	F4—B1—F2	108.7 (2)
C8—C13—H13A	119.8	F3—B1—F2	109.0 (2)
N2—C14—C11	112.09 (17)	F4—B1—F1	109.9 (2)
N2—C14—H14A	109.2	F3—B1—F1	102.7 (2)
C11—C14—H14A	109.2	F2—B1—F1	109.9 (2)
			
C7—C2—C3—C4	0.8 (3)	C9—C10—C11—C14	179.33 (19)
C1—C2—C3—C4	179.9 (2)	C10—C11—C12—C13	−0.8 (3)
C2—C3—C4—C5	0.1 (3)	C14—C11—C12—C13	179.01 (18)
C3—C4—C5—C6	−1.1 (4)	C11—C12—C13—C8	1.3 (3)
C4—C5—C6—C7	1.2 (3)	C9—C8—C13—C12	−0.2 (3)
C5—C6—C7—C2	−0.3 (3)	C7—C8—C13—C12	179.14 (18)
C5—C6—C7—C8	−177.10 (19)	C12—C11—C14—N2	114.6 (2)
C3—C2—C7—C6	−0.7 (3)	C10—C11—C14—N2	−65.7 (3)
C1—C2—C7—C6	−179.81 (19)	N2—C16—C17—O1	58.1 (3)
C3—C2—C7—C8	176.01 (18)	O1—C18—C19—N2	−56.8 (3)
C1—C2—C7—C8	−3.1 (3)	C17—C16—N2—C19	−53.4 (2)
C6—C7—C8—C9	54.6 (3)	C17—C16—N2—C14	−177.59 (18)
C2—C7—C8—C9	−122.0 (2)	C18—C19—N2—C16	52.3 (2)
C6—C7—C8—C13	−124.7 (2)	C18—C19—N2—C14	176.95 (18)
C2—C7—C8—C13	58.6 (3)	C11—C14—N2—C16	−64.1 (2)
C13—C8—C9—C10	−1.5 (3)	C11—C14—N2—C19	172.46 (17)
C7—C8—C9—C10	179.17 (19)	C19—C18—O1—C17	61.2 (2)
C8—C9—C10—C11	2.0 (3)	C16—C17—O1—C18	−61.4 (3)
C9—C10—C11—C12	−0.9 (3)		

### Hydrogen-bond geometry (Å, °)

**Table:**

*D*—H···*A*	*D*—H	H···*A*	*D*···*A*	*D*—H···*A*
N2—H2···F1	0.90 (2)	2.14 (2)	2.902 (3)	141 (2)
N2—H2···F3	0.90 (2)	2.35 (2)	3.219 (3)	161 (2)
